# Robust discrimination between EEG responses to categories of environmental sounds in early coma

**DOI:** 10.3389/fpsyg.2014.00155

**Published:** 2014-02-25

**Authors:** Natacha Cossy, Athina Tzovara, Alexandre Simonin, Andrea O. Rossetti, Marzia De Lucia

**Affiliations:** ^1^Electroencephalography Brain Mapping Core, Center for Biomedical Imaging (CIBM), University Hospital Center, University of LausanneLausanne, Switzerland; ^2^Department of Radiology, University Hospital Center, University of LausanneLausanne, Switzerland; ^3^Department of Clinical Neurosciences, University Hospital Center, University of LausanneLausanne, Switzerland

**Keywords:** auditory processing, voice, environmental sounds, coma, multivariate decoding, single-trial EEG

## Abstract

Humans can recognize categories of environmental sounds, including vocalizations produced by humans and animals and the sounds of man-made objects. Most neuroimaging investigations of environmental sound discrimination have studied subjects while consciously perceiving and often explicitly recognizing the stimuli. Consequently, it remains unclear to what extent auditory object processing occurs independently of task demands and consciousness. Studies in animal models have shown that environmental sound discrimination at a neural level persists even in anesthetized preparations, whereas data from anesthetized humans has thus far provided null results. Here, we studied comatose patients as a model of environmental sound discrimination capacities during unconsciousness. We included 19 comatose patients treated with therapeutic hypothermia (TH) during the first 2 days of coma, while recording nineteen-channel electroencephalography (EEG). At the level of each individual patient, we applied a decoding algorithm to quantify the differential EEG responses to human vs. animal vocalizations as well as to sounds of living vocalizations vs. man-made objects. Discrimination between vocalization types was accurate in 11 patients and discrimination between sounds from living and man-made sources in 10 patients. At the group level, the results were significant only for the comparison between vocalization types. These results lay the groundwork for disentangling truly preferential activations in response to auditory categories, and the contribution of awareness to auditory category discrimination.

## Introduction

Intense research activity in recent years has focused on the neural mechanisms underlying the ability to recognize environmental sounds, including vocalizations, the sounds of tools, and the sounds of musical instruments (Belin et al., 2000; Levy et al., 2001; Lewis et al., 2005; Murray et al., 2006; Staeren et al., 2009; De Lucia et al., 2010a; Giordano et al., 2013). The vast majority of these studies investigated the neural correlates of such discrimination while subjects could consciously perceive and recognize each sound category, with few exceptions (Plourde et al., 2006; De Lucia et al., 2012). As a result, it remains controversial whether the categorization of environmental sounds occurs independently of consciousness, and, by extension, overt behavior. Understanding whether this auditory processing survives in the absence of consciousness can help in establishing the existence of category-sensitive regions irrespective of the subject's ability to attend to and recognize specific sounds. One example of this debate concerns the existence of voice-specific responses as measured by electroencephalography (EEG). Levy et al. reported the existence of a voice-specific response, which was only evident when subjects were explicitly attending to the stimuli (Levy et al., 2001, 2003). More recent electrophysiological studies (Murray et al., 2006; Charest et al., 2009; De Lucia et al., 2010a) have provided evidence of significant differential activity in response to auditory categories, including vocalizations, despite the task-irrelevance of the categorization. Because the role of attention and consciousness in processing sensory stimuli can overlap and interact (Kiefer and Martens, 2010; van Boxtel et al., 2010; Kiefer, 2012), in the present study we contribute to this ongoing debate by assessing whether auditory discrimination of environmental sounds can be achieved in the absence of consciousness.

Specifically, we address this question by analyzing the EEG response to environmental sounds in a group of comatose patients. To the best of our knowledge no studies have so far reported evidence in favor of the existence of environmental sound discrimination in the absence of consciousness in humans. Previous evidence in humans is consistent with the existence of an implicit mechanism supporting auditory processing of environmental sounds. Functional magnetic resonance imaging (fMRI) studies have shown that categorical discrimination between environmental sounds involves auditory-responsive cortices along the superior and middle temporal gyri, including primary and secondary cortices that are typically considered responsive to low-level acoustic features (Formisano et al., 2008; Staeren et al., 2009; Leaver and Rauschecker, 2010). The temporal dynamics of these processes as revealed by electroencephalographic studies indicate that categories of environmental sounds are discriminated at early (i.e., <200 ms) post-stimulus latencies and within middle temporal cortices (Murray et al., 2006; De Lucia et al., 2010a). Another recent electroencephalographic study provided evidence of discrimination between auditory categories even when subjects could not recognize the sounds, which unfolded over two distinct temporal stages; one that was related to implicit auditory processing and the other that was linked to accurate categorical perception (De Lucia et al., 2012).

Additional support for the existence of an implicit mechanism underlying auditory representation of environmental sounds comes from animal research. Differential responses to conspecific vocalizations in superior temporal cortices have been observed despite anesthesia (Rauschecker et al., 1995; Tian et al., 2001; Wang and Kadia, 2001; Petkov et al., 2008). In awake preparations, responsive cortices expand to include pre-frontal regions (Poremba et al., 2004; Cohen et al., 2007; Russ et al., 2008; Romanski and Averbeck, 2009). In humans, auditory processing in the absence of consciousness can be studied in patients with disorders of consciousness, under anesthesia and during sleep (Chennu and Bekinschtein, 2012). Neural processing of sound concepts in the absence of consciousness can also be studied by using masked priming paradigms (Trumpp et al., 2013a). More specifically in the context of auditory discrimination of environmental sounds, one attempt has been reported in an fMRI study (Plourde et al., 2006) where subjects exhibited activations in response to complex auditory stimuli during propofol-induced anesthesia but failed to show any category-specific activation.

Here we carried out an auditory evoked potential (AEP) study in post-anoxic comatose patients during the very early phase of coma. All patients were recorded twice: the first time while under therapeutic hypothermia (TH) and sedated, hence unconscious, the second time while brought back to normal temperature. We presented comatose patients with series of human and animal vocalizations, and sounds from man-made objects, while recording EEG. We applied a multivariate decoding algorithm at the level of the single-trial EEG responses to sounds (Tzovara et al., 2012b); a method previously applied for decoding categories of environmental sounds in healthy subjects (De Lucia et al., 2012) and for tracking the progression of auditory discrimination during a mismatch negativity (MMN) paradigm in comatose patients (Tzovara et al., 2013; Rossetti et al., in press). This method allows for the identification of spatio-temporal patterns of differential EEG responses to categories of sounds and the quantification of the degree of auditory discrimination at the level of the single subject, which is particularly suitable for clinical populations where one can expect a high degree of inter-patient variability.

## Methods

### Patients and controls

The experiment included 22 post-anoxic comatose patients (three women; mean age ± standard error 65.55 ± 0.60 years, range, 45–87 years). They had been admitted from November 2011 to July 2012 to the Department of adult Critical Care Medicine at the Centre Hospitalier Universitaire Vaudois (CHUV) in Lausanne. The study was approved by the Ethics Committee of the Faculty of Biology and Medicine of the University of Lausanne.

On the basis of the Glasgow Coma Scale and the “Four Score” scale (Wijdicks et al., 2005; Bruno et al., 2011), the level of consciousness was evaluated every 2–3 h during the first 48 h after coma onset. All patients scored 3 or 4 in the Glasgow Coma Scale during both recordings, corresponding to a deep unconscious state. All patients were cared for according to a standard protocol (Oddo et al., 2006) after being resuscitated following current recommendations (2005 American Heart Association Guidelines for Cardiopulmonary Resuscitation and Emergency Cardiovascular Care Circulation 2005:112:IV1-203). After resuscitation, patients were cooled during 24 h to 33°C by using ice-packs, intravenous ice-cold fluids and a surface cooling device (Arctic Sun System, Medivance, Louisville, CO, USA), which allows the maintenance of TH. During TH, vecuronium (0.1 mg/kg boluses) was administrated to control shivering and midazolam (0.1 mg/kg/h) and fentanyl (1.5 μg/kg/h) were used for sedation. Patients with myoclonus and/or status epilepticus received non-sedative intravenous antiepileptic drugs (mostly levetiracetam, valproate), which were discontinued if no clinical improvement was observed after at least 72 h.

A decision on withdrawal of intensive care support was based on two of the following criteria: incomplete recovery of brainstem reflexes, early myoclonus, bilateral absence of somatosensory evoked potentials (SSEPs) and non-reactive EEG background to stimulation (Rossetti et al., 2010). All these criteria were assessed in normothermia (NT), at least 48–72 h after cardiac arrest and off sedation. Survival was evaluated at 3 months.

In total, 22 patients were recorded but three were disregarded because of too few artifact-free trials (i.e., less than 60). Nevertheless, all the others were included without any *a priori* selection based on the presence of an ERP. In addition to the 22 patients, we recorded EEG from 10 control subjects (6 women; mean age 57.7 ± 2.3 years, range 47–73 years) in order to assess the degree of decoding accuracy in a healthy population in the same age range as the comatose patients and with the same protocol and setup. None of the control subjects reported hearing problems or history of psychiatric or neurological illnesses. For comatose patients, we could not ensure that they had no hearing or pre-existing neurological problems.

### Stimuli

**Figure 1:**
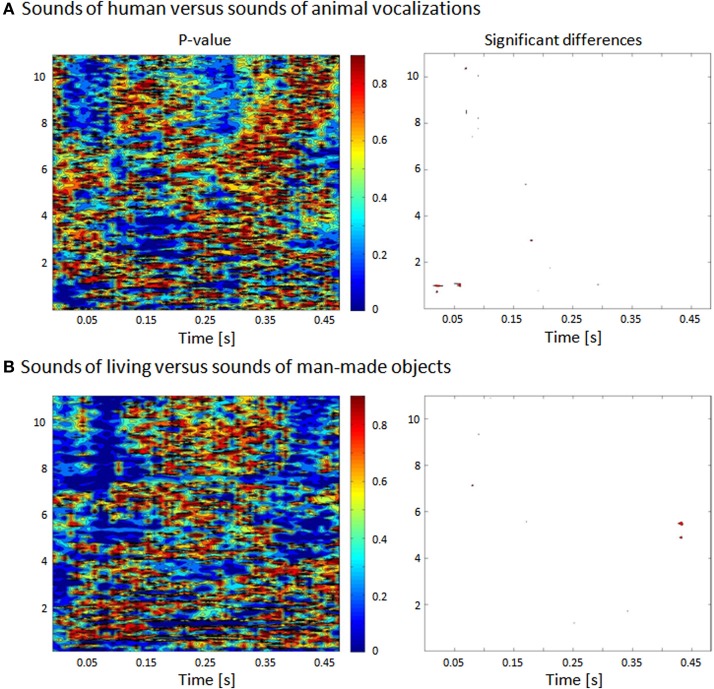
**Statistical comparison between human and animal vocalizations and between living and man-made sounds based on their frequency. (A)** The spectrogram of each sound was generated and comparisons (nonparametric *t*-tests) were made across groups of stimuli for each 5 ms and 160 Hz time-frequency bin. **(B)** Bins reaching the statistical criterion of *p* < 0.05 are displayed in red.

Auditory stimuli were complex, meaningful sounds (16 bit stereo; 22,500 Hz digitization) including human and animal vocalizations as well as sounds of man-made objects. In the following we will refer to the category including all the vocalizations as that of sounds of living objects. These sounds have been previously used in prior behavioral, EEG, and fMRI studies (Murray et al., 2006; De Lucia et al., 2009, 2010a,b). Living sounds included 12 animal vocalizations (sheep, rooster, pig, owl, frog, donkey, dog, crow, chicken, cow, cat, and birds) and eight human sounds (whistling, sneezing, screaming, laughing, gargling, coughing, clearing one's throat, and crying). None of these human sounds contained verbal information. Man-made sounds were an accordion, bicycle bell, car horn, cash register, church bell, cuckoo clock, doorbell, door closing, flute, glass shattering, guitar, harmonica, harp, organ, piano, police siren, saxophone, telephone, trumpet, and violin. Each sound was 500 ms duration and was normalized according to the root mean square of their amplitude. In order to exclude that differential activity in response to the categories we consider in this study could be explained by simple acoustic differences, we compared the group of sounds including human vocalizations vs. the animal vocalizations with respect to several features as explained in the following. We repeated the same analysis in the comparison between all the vocalizations and the man-made sounds. In previous studies we had already analyzed the possible difference in terms of formants between the human and animal vocalizations (De Lucia et al., 2010a). The groups of sounds were compared in terms of their acoustic properties by assessing their spectrogram (Figure 1), their mean harmonics-to-noise ratio (HNR) and their power spectrum (Murray et al., 2006; De Lucia et al., 2010a); no significant differences were observed.

The acoustic analysis between the spectrograms (defined with Matlab's spectrogram function with no overlapping and zero padding) of human and animal vocalizations sounds was based on a non–parametric statistical comparison, using a time–frequency bin width of 5 ms and 74 Hz. Specifically the statistical contrasts comprised a series of nonparametric *t*-tests based on a bootstrapping procedure with 5000 iterations per time–frequency bin in order to estimate an empirical distribution against which to compare the actual difference between the mean spectrograms from each sound category (Knebel et al., 2008). A significant difference at a given time–frequency bin was only accepted if all eight of its immediately adjacent bins also yielded values of *p* < 0.05 (i.e., a 3 × 3 bin spatial threshold was applied).

### Procedure and task

Sounds of living and man-made objects were delivered in a pseudo-random order to patients via insert earphones (Etymotic model: ER4S). Patients heard four blocks of 3 min, each containing 20 man-made sounds and 20 living sounds repeated twice, resulting in a total of 80 trials per block. Concretely, 160 trials were available for both living (96 and 64 for animal and human vocalizations, respectively) and man-made sounds. The sounds were delivered with an inter-stimulus interval of 2 ± 0.2 s to minimize anticipation.

### EEG acquisition and preprocessing

We performed continuous EEG recording (Viasys Neurocare, Madison, WI, USA) during TH and NT with 19 electrodes placed according to the international 10–20 system (sampling rate at 1024 Hz). The impedances of all electrodes were maintained below 10 kΩ. All the EEGs were recorded at bedside, in the clinical environment and without disturbing the clinical routine. In particular, the current experiment was run just after or before the clinical EEG recording using the same setup. For reasons of consistency, EEG recordings on control subjects were performed with the same procedure and equipment. Healthy subjects, while lying on an inclined chair in a hospital room, were asked to keep their eyes closed and to listen to the sounds.

Peri-stimulus EEG epochs were extracted, spanning 100 ms before stimulus onset up to 800 ms after sound onset. The data were filtered with 0.1–40 Hz band-pass and 50 Hz notch. An artifact rejection criterion of ±100 μV was applied offline to each channel, and no baseline correction was performed. After EEG preprocessing three patients were excluded because they did not have enough trials for each condition (less than 60 trials). Among the remaining 19 patients we could extract enough artifact-free trials in response to human and animal sounds for 17 patients in TH and 18 patients in NT (35 comparisons in total).

Within these 19 patients, an average of 3 ± 1 and 4 ± 1% of the trials in response to animal and human vocalizations, respectively, were rejected for the recording during TH. For the EEG recording during NT, 5 ± 1 and 4 ± 1% of the trials in response to animal and human voices, respectively, were rejected. These values did not differ between conditions (i.e., animal/human) or recordings (i.e., TH/NT) (*t*-test; *p* > 0.05).

Concerning the trials in response to living and man-made sounds, an average of 3 ± 1 and 2 ± 1% of the trials, respectively, were rejected for the recording in TH. For the EEG recording in NT, 5 ± 1 and 5 ± 1% of the trials in response to living and man-made respectively were rejected. These values did not differ between conditions (i.e., living/man-made) or recordings (i.e., TH/NT) (*t*-test; *p* > 0.05).

### Multivariate EEG decoding

In order to assess the difference in brain responses to living vs. man-made auditory objects or human vs. animal vocalizations, we used a multivariate EEG decoding approach (Tzovara et al., 2012a,b). This method is based on modeling single-trial EEG voltage topographies in order to identify temporal periods of differential EEG activity at the single-patient level. We applied the analysis to each of the recordings and compared separately the EEG responses to living vs. man-made sounds and human vs. animal vocalizations.

### Datasets definition

For each sounds category, the whole set of available single trials was divided in cross-validation (CV) dataset and validation (V) dataset. As it will be clear in the following, the first of these sets will be used for model training and testing, the V dataset will be used for providing an independent estimation of the decoding performance. The separation between the CV and V datasets is required because the CV dataset is exploited for selecting the algorithm's parameters that best discriminate between single trials in response to different sounds categories (i.e., the total number of topographies). A realistic estimation of the decoder performance needs to be evaluated on a separate set of data (i.e., the V dataset) from what is used for model selection.

In all the analyses performed at single-patient/single-subject level, the number of trials included in the CV was always 60 per condition in all the comparisons between EEG responses to human and animal vocalizations, and those to sounds of living and man-made objects. The rest of the trials were included in the V dataset. The trials assigned to CV and V datasets were extracted randomly within all the accepted trials for each recording. This random assignment minimizes the effect of any systematic variation along the recordings on the decoding performance obtained on the validation dataset. As explained above, because of this constraint of having 60 artifact-free trials in the CV dataset, we excluded three patients because of the presence of artifacts. Among the remaining 19 patients we could extract enough artifact-free trials in response to human and animal sounds for 17 patients in TH and 18 patients in NT (35 comparisons in total). For reasons of consistency we also restricted our analyses of responses to living vs. man-made sounds to the same patients and recordings (i.e., TH/NT).

In the healthy controls we had a total of eight subjects who had at least 60 artifact-free trials in the CV dataset for each of the conditions to be compared. However, for the analysis across all the control subjects we included all the ten subjects. Specifically in the group analysis we considered 200 trials in the CV (20 trials for each subject) and 40 trials for each condition in the V dataset (four trials for each subject).

### Multivariate decoding analysis: cross-validation

The technique consists in the decoding of stimulus categories at the single-trial level. It involves the modeling of voltage topographies of the single-trial AEPs by a Mixture of Gaussians (GMM). Each patient and each of the two recording datasets (i.e., TH and NT) were analyzed separately. The procedure is divided into the CV and the validation (V) phase, each of them involving the CV and V datasets as defined above.

The CV consists of model training and testing and aims at obtaining a model that allows an optimal discrimination of EEG responses to different sound categories. The model is based on a Mixture of Gaussians (GMM, Dempster et al., 1977) in a number of dimensions which equals the number of electrodes of the EEG recording. The GMM parameters estimation is based on the ensemble of instantaneous voltage topographies, from all latencies and trials (see Figure 5 in Tzovara et al., 2012a). The decoding algorithm takes into account the mean voltage topographies (template maps) for each gaussian in the GMM and the period of time, H, at which these template maps mostly differ between the two conditions of interest (see Figure 6 in Tzovara et al., 2012a). Following a standard procedure in decoding analysis (Pereira et al., 2009), this model estimation is performed on one part of the trials of the CV dataset (training).

**Figure 2:**
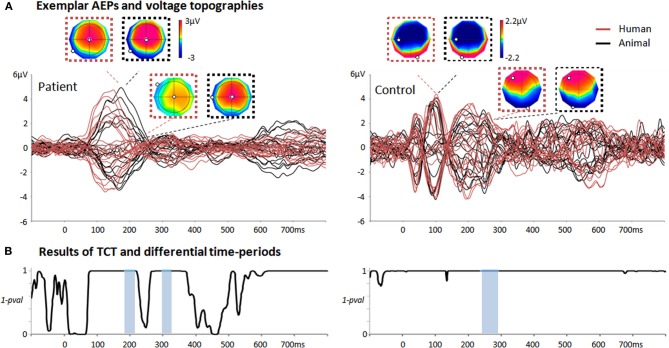
**Summary of the single-trial EEG analysis when comparing responses to animal/human vocalizations in two exemplar individuals. (A)** Average auditory evoked potentials of one patient recorded during TH and one control subject in response to human (red) and animal (black) vocalizations. For the patient, the voltage topographies correspond to the first peak of GFP and to the first period of differential activity as evaluated by the single-trial EEG analysis (184–214 ms post-stimulus onset). For the control, the first two voltage topographies correspond to the N100; the second two correspond to the voltage topographies occurring during the period of differential activity identified by the single-trial topographic analysis (237–295 ms post-stimulus onset). The N100 voltage topographies exhibit a prototypical distribution in classical AEP responses (in contrast to the maps of the patient at the same latencies). **(B)** Results of the TCT for the patient and control subject revealing long-lasting time-periods of evoked responses (i.e., periods of 1-*p* > 0.999). Periods of differential activity in response to animal and human vocalizations estimated by the single-trial decoding analysis are highlighted in light blue.

**Figure 3:**
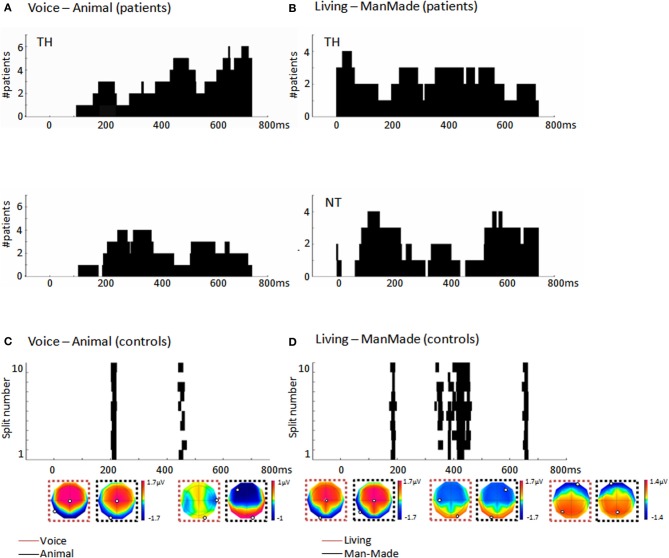
**(A)** Histograms (40 ms bins) of the time-periods of differential activity (H, cf. section “Multivariate EEG decoding”) in response to different vocalization types as computed by the single-trial decoding analysis across the significant patients. Top panel: During TH for the human/animal comparison, the most consistent period was at ~450 ms post-stimulus onset, observed in five out of the seven significant patients. Bottom panel: During NT half of the patients showed earlier discriminative periods at ~300 ms **(B)** Histograms (40 ms bins) of the time-periods of differential activity, H, in response to living and man-made sounds as computed by the single-trial decoding analysis across the significant patients. Top panel: During TH, the living/manmade comparisons provided a consistent period of differential activity starting already at ~50 ms post-stimulus onset in four out of the five patients; Bottom panel: During NT four patients out of six had a consistent period of differential activity between living and man-made sounds around 180 ms post-stimulus onset as well as a later period after 600 ms post-stimulus. **(C)** Results of the single-trial decoding analysis applied across the group of 10 control subjects and comparing EEG responses to human and animal vocalizations. Each line provides the time period of differential activity, H, in each split of the cross-validation procedure. The first of these time-periods overlapped with what observed in patients during NT around 200 ms. **(D)** Time periods of differential activity, H, identified by the single-trial decoding analysis across the 10 control subjects when comparing EEG responses to living and man-made sounds. Each line represents the result obtained in each split of the cross-validation. The first of this differential period at 173 ms pos-stimulus onset overlaps with what observed in patients during NT. Mean voltage topographies along the time periods of differential activity are shown for each of the categories (minimum and maximum values are highlighted in each topography).

In the model testing, trials of the remaining part of the CV dataset are assigned to one of the two sets of template maps. In other words, using the representative template maps extracted along H, we decode the category of sounds that was presented to the patient on every trial. Examples of the estimated discriminative time-periods H can be seen in Figure 2 (highlighted in light blue), and in Figure 3 for all the patients and the control group. The decoding performance was based on the area under the Receiving Operating Characteristic (Green and Swets, 1966) when classifying EEG responses to different sound categories. The abovementioned procedure is repeated with different number of Gaussians in the GMM in order to find an optimal model, namely the one maximizing the area under the Receiver Operating Characteristic (AUC) across test datasets. The whole CV procedure is repeated six times, by considering a different part of the data as test dataset, in a way that the six test datasets do not overlap.

### Multivariate decoding analysis: validation and significance assessment

In the V phase, we used the model selected during the CV phase to classify trials of an independent and separate dataset (V dataset) in one of the two sounds categories. In particular, the V dataset was never used for selecting the models' parameters. To evaluate the significance of the AUC values obtained in the V dataset, we ran a permutation test. This test consists in randomly permuting the trials belonging to the two experimental conditions of the CV datasets and evaluating two new GMM models for each permutation. The permutation is done 200 times. These two sets of models are used for classifying the trials of the validation dataset. The distribution of the decoding accuracy obtained with the permuted trials is compared to the decoding accuracy obtained with the true trials' partition. This step aims at verifying that the decoding accuracy obtained in V is better than expected by chance. We considered classification accuracy significant if the true AUC based on the validation dataset outperformed the ones obtained on the random models by applying a Wilcoxon signed rank test (*p* < 0.001). This test is commonly used for assessing the significance of classification performance in neuroimaging studies (Pereira et al., 2009). We report in the following the classification accuracy measured in the V datasets for those patients and controls for which results were significant. In addition, we performed the same decoding analysis at the group level only for the control subjects similarly to what has been already reported in De Lucia et al. (2012).

We evaluated the significance of our decoding results across all patients using a similar approach as in previous EEG decoding studies (Hausfeld et al., 2012). We used a binomial test (using the Matlab function binocdf), with *n* = total number of comparisons and *k* the number of patients showing significant decoding results. The probability of significance for each event was assessed based on permutation, that is to say by evaluating the number of times the decoding performance obtained on the validation dataset outperformed that obtained on random permutation. This test provides an estimation of the probability to observe by chance significant results in *k* out of *n* tests (here *n* = 35; Supplemental Figure).

### Topographic consistency test (TCT)

Typically, AEPs recorded in patients can exhibit very different patterns than those recorded in healthy subjects under the same conditions. Consequently, assessing the presence of an evoked response is particularly challenging in this context. In a classical approach, the assessment of a robust ERP in response to sounds is based on the presence of a significant voltage amplitude modulation at a pre-selected scalp location at about 100 ms post-stimulus onset (Fischer et al., 1999b). Previous studies have systematically disregarded a high percentage of patients because of the absence of a typical N100 (Fischer et al., 1999b). However, due to the pathological conditions of these patients, recorded ERPs can fail the test only because they do not exhibit prototypical waveforms or appear at different latencies (see Figure 2A, left panel for an exemplar patient).

In the present study, we assessed the presence of an evoked response by taking advantage of voltage topographies and their repetition across trials at a fixed latency (Koenig and Melie-Garcia, 2010), without pre-selection of the latency at which a specific EEG component is presumed to appear. Specifically, TCT allows computing time-periods during which the voltage topography is consistent across repeated measurements in event-related responses at any latency from stimulus onset. This test allows quantifying the presence of an evoked response and is more adapted to our analysis than looking at the N100 component, as it is based on the voltage topography and not on a single electrode.

TCT is assessed by measuring the Global Field Power (GFP; Koenig and Melie-Garcia, 2010) and a randomization procedure that involves the random shuffling, of each time point from each trial, of data across electrodes. The randomized values at one time frame are then averaged across trials, leading to a mean topographic map and GFP. By repeating this randomization procedure many times, we obtain an empirical distribution of the GFP of an average voltage map. We then compare the GFP value of the average map from the real data with the empirical distribution in order to compute the number of times that the real GFP is lower than those obtained after the randomization. To find statistical periods of topographic consistency, a correction for multiple testing was applied to re-estimate the threshold of the p-value; it is based on the False Discovery Rate, (Genovese et al., 2002), which controls the expected number of incorrectly rejected hypotheses. TCT was performed for each subject and each experimental condition, separately.

## Results

### Auditory discrimination in comatose patients

For each patient and each recording, we compared responses to living and man-made sounds and to human and animal vocalizations, separately. Eleven out of nineteen patients showed significant discrimination between animal and human vocalizations (Wilcoxon signed-rank test; *z* ≤ −7.42; *p* < 0.001). Among them, four were significant only during TH and four only during NT (the remaining three patients showed significant results in both TH and NT). The mean AUC across the seven patients exhibiting significant decoding performance during TH was 0.63 ± 0.03 (0.53 ± 0.01 was the mean AUC value at chance level). The seven patients who showed significant discrimination between animal and human vocalizations during NT had a mean AUC of 0.65 ± 0.05 (0.49 ± 0.07 was chance level). A summary of these results is shown in Table 1.

**Table 1:** **Summary of the decoding performance results for each of the categorical comparison, voice-animal and living-manmade and each recording during TH and NT**.

	**No. of significant patients**		**Decoding value (s.e.m.)**		**Chance level (s.e.m.)**	
	**TH**	**NT**	**TH**	**NT**	**TH**	**NT**
Voice—animal	7	7	0.63 (0.03)	0.65 (0.05)	0.53 (0.01)	0.49 (0.07)
Living-manmade	5	6	0.60 (0.02)	0.63 (0.05)	0.54 (0.03)	0.57 (0.05)

*The total number of patients exhibiting significant results is shown. Some of these patients had significant results both during TH and during NT (four for the Voice-Animal comparison, and one for the Living-Manmade one). The mean decoding value and the chance level (standard error in parentheses) are computed across the significant results only*.

The binomial test across all patients was used to estimate the minimum number of patients that should have significant decoding results, to consider our results significant at the level of the group. This test gave a low probability (*p*_group_ < 0.05) of observing significant results by chance in 12 or more out of 35 comparisons (Supplemental Figure). We therefore considered significant at the group level the decoding accuracy of the voice/animal comparison, because we obtained significant results at single recording level in more than 12 recordings.

The same analysis was repeated for comparing responses to living and man-made sounds. In total we observed significant decoding performance in 10 patients (Wilcoxon signed rank test, *z* ≤ −4.80, *p* < 0.001), within whom four only during TH and five during NT only (one patient had significant results during TH and NT). The mean decoding performance across significant results in V during TH was 0.60 ± 0.02 (0.54 ± 0.03 was chance level). During NT we obtained a mean decoding performance of 0.63 ± 0.05 (0.57 ± 0.05 was chance level). Importantly, these results were obtained based on the same number of single trials in the CV and V as those included in the analysis for decoding EEG responses to human and animal vocalizations. A summary of the decoding results for is shown in Table 1. In this case the binomial test at the group level showed that the probability of observing significant results in 10 recordings out of 35 was greater than 0.05. Therefore, these results could not be considered significant at the group level at least by assuming that our AUC values were following a binomial distribution. Despite this analysis providing non-significant results, we will nevertheless discuss them for two reasons. First, the percentage of patients exhibiting significant results at the single-patient level is similar to what has been reported in the literature on semantic categorization in comatose patients (Fischer et al., 2008; Daltrozzo et al., 2009). Second, the analysis relies on assuming that the AUC values follow a binomial distribution, which remains an untested hypothesis. In light of these points, our results show that both types of auditory categorization provided significant results at the single-patient level (in a subset of individuals). The vocalization comparison also provided significant results at the group level based on the hypothesis that the AUC values follow a binomial distribution. In addition, we investigated whether or not significant auditory discrimination was linked to clinical variables such as patients' outcome and time to return of spontaneous circulation (ROSC), or age and time of EEG recordings. When comparing all these values between patients exhibiting significant decoding performance and those without significant discrimination of vocalization types, we found no significant differences [Table 2; unpaired *t*-tests, |*t*_(17)_| ≤ 0.93, *p* ≥ 0.36]. The same analysis performed on those patients who exhibited significant decoding between EEG responses to living and man-made sounds provided similarly non-significant results [Table 3; unpaired *t*-tests, |*t*_(17)_| ≤ 1.69, *p* ≥ 0.11]. Moreover, significant discrimination between auditory categories was not associated with awakening from coma in either case (Tables 2, 3 first lines). Finally, we did not find any relation between the progression of auditory discrimination and patients outcome (Tzovara et al., 2013).

**Table 2:** **Description of the patients, separated according to whether they exhibited vocalizations discrimination (*n* = 11 patients, left column) or not (*n* = 8 patients, right column)**.

	**Patients with discrimination (*n* = 11)**	**Patients without discrimination (*n* = 8)**	***p*-value**
Patients alive at 3 months (%)	55	63	
Age (years)	67 ± 4	63 ± 5	0.48
Time to ROSC (min)	18 ± 3	21 ± 4	0.49
Time to 1rst EEG (h)	18 ± 2	18 ± 1	0.97
Time between recordings (h)	28 ± 3	25 ± 1	0.36

*Patients are described regarding the proportion of survivors at 3 months after coma onset, their mean age, mean time of cardiac arrest (i.e., time from circulatory arrest to return of spontaneous circulation, ROSC), mean time between coma onset and the first EEG, and mean time between the two recordings (TH and NT). T-values: |t(17)| ≤ 0.93*.

**Table 3:** **Description of the patients, separated according to whether they exhibited discrimination between living and man-made objects (*n* = 10 patients, left column) or not (*n* = 9 patients, right column)**.

	**Patients with discrimination (*n* = 10)**	**Patients without discrimination (*n* = 9)**	***p*-value**
Patients alive at 3 months (%)	30	89	
Age (years)	65 ± 5	66 ± 3	0.82
Time to ROSC (min)	23 ± 3	15 ± 3	0.11
Time to 1rst EEG (h)	19 ± 1	17 ± 2	0.44
Time between recordings (h)	27 ± 2	26 ± 2	0.9

*Patients are described regarding the proportion of survivors at 3 months after coma onset, their mean age, mean time of cardiac arrest (i.e., time from circulatory arrest to return of spontaneous circulation, ROSC), mean time between coma onset and the first EEG, and mean time between the two recordings (TH and NT). T-values: |t| ≤ 1.69*.

### Periods of differential activity between auditory categories in patients

Among the seven patients exhibiting a discrimination of animal and human vocalizations during TH, five showed a consistency in the latencies of differential activity at about 450 ms post-stimulus onset although the vast majority of the patients had differential activity after 700 ms (Figure 3A). During NT four patients showed earlier discriminative periods at ~300 ms (Figure 3A). The comparison between EEG responses to living and man-made sounds revealed consistent periods of differential activity starting at ~50 ms post-stimulus onset in four that were significant during TH (Figure 3B). During NT four out of the six patients had a consistent period of differential activity between living and man-made sounds around 180 ms post-stimulus onset as well as a later period around 600 ms post-stimulus. Irrespective of being recorded during TH or NT, the percentage of patients showing significant discrimination between living and man-made sounds exhibited earlier latencies of differential activity than when processing different vocalizations type, a result which is in accord with previous evidence in healthy participants (De Lucia et al., 2010a).

### Topographic consistency test

The TCT test revealed that the majority of patients had periods of consistent evoked responses throughout the whole trial. In particular, 18 patients in TH and 16 in NT had consistent periods in response to living sounds, while 16 patients in TH and 17 in NT had such periods in response to man-made sounds (Figure 2A left panel for one exemplar patient during NT). However, not all these periods of responses overlapped with the classical latency for an N100 component (100 ± 50 ms). The TCT was also applied to the 10 control subjects and revealed that all of them had a topographic consistency at 100 ± 50 ms, corresponding to the latency of a classical N100 component (Figure 2B right panel for an exemplar subject).

### Auditory discrimination in healthy controls

To compare the level of neural discrimination of comatose patients with that of healthy subjects, we additionally applied the same procedure of data analysis to the 10 age-matched healthy subjects. We obtained significant decoding accuracy in two controls when comparing responses to human and animal vocalizations (out of the eight who had enough artifact-free trials); in the comparison between responses to living and man-made sounds we obtained four controls who exhibited accurate decoding, one of whom also had significant results in the previous comparison. The average decoding performance was 0.64 ± 0.12 (Wilcoxon signed-rank test; *z* ≤ −9.26; *p* < 0.001) and 0.64 ± 0.01, respectively, for the comparison between vocalizations and that between responses to living and man-made sounds (Wilcoxon signed-rank test; *z* ≤ −3.30; *p* < 0.001). The analysis at the single-trial level and across all the 10 subjects (by assessing one GMM model across subjects for each condition) revealed a significant categorization at the neural level between vocalization types with a decoding performance of 0.58, being 0.56 the average decoding accuracy at chance level (Wilcoxon signed-rank test; *z* = −5.48; *p* < 0.001). When trying to decode EEG responses between living and man-made sounds we also obtained significant discrimination across the 10 control subjects, with an AUC of 0.60 and 0.56 the average decoding accuracy at chance level (Wilcoxon signed-rank test; *z* = −9.21; *p* < 0.001). The periods of differential responses obtained in this group analysis occurred at 200 and 173 ms post-stimulus onset for the vocalization types and living/man-made comparisons respectively (Figure 3). These two periods overlapped with the first differential periods observed in most of the patients during NT.

## Discussion

This is the first study to report evidence of auditory discrimination of environmental sounds in early phases of coma, even under sedation and during TH. In particular, we observed robust discrimination between vocalization types in 11 out of 19 patients, and more general discrimination between sounds from living and man-made sources in 10 out of 19 patients. Our results provide evidence of auditory discrimination without any overt awareness of the external stimuli, showing that discrimination between categories of environmental sounds relies, at least in part, on automatic and implicit auditory processing.

### Comparison to previous literature

An extensive literature based on fMRI (Fecteau et al., 2004; Lewis et al., 2005; Formisano et al., 2008; Bestelmeyer et al., 2011; Giordano et al., 2013), electrophysiological recordings in monkeys (Tian et al., 2001; Poremba et al., 2004; Petkov et al., 2008; Recanzone, 2008; Romanski and Averbeck, 2009; Perrodin et al., 2011) and electrophysiological studies in humans (Levy et al., 2003; Murray et al., 2006; Charest et al., 2009; De Lucia et al., 2010a) has investigated the extent of categorical discrimination in the auditory modality. In particular the study of the neural correlates of voice discrimination has emphasized the existence of brain regions that activate when discriminating conspecific vocalizations in humans vs. other categories, which is located mainly in the right superior temporal sulcus and extends into the superior temporal gyrus (Belin et al., 2000; Fecteau et al., 2004; De Lucia et al., 2010a).

However, no study in humans has provided evidence of neural discrimination of auditory categories in the absence of consciousness. Consequently, it remained unresolved how the neural correlates of auditory discrimination of environmental sounds are influenced by task demand and the subject's ability to recognize the auditory object. During active tasks, the subject's attention has been shown to play a crucial role in accurate sound discrimination (Levy et al., 2001, 2003). A systematic investigation of the neural correlates of auditory semantic processing as a function of sound recognizability, attention, and tasks is a crucial step in identifying the role of specific areas and activations along the so-called “what” pathway (Rauschecker and Tian, 2000; Clarke et al., 2002) implicated in auditory object representations. One important step toward this ambitious goal is assessing whether auditory categorization of environmental sounds is possible in a condition when any form of conscious access to auditory sensory stimuli can be excluded.

To the best of our knowledge, the only attempt to reveal neural correlates of representations of environmental sounds in humans in the absence of consciousness has been reported in an fMRI study under propofol-induced anesthesia (Plourde et al., 2006). Even though stimuli, including spoken words, scrambled words, and other vocalizations, could elicit significant activations with respect to baseline, anesthesia abolished any word-specific activation. In this previous study anesthesia was necessary to ensure unconsciousness in healthy controls. In our case, patients received a lighter dose of sedation and their unconsciousness was instead the result of the lack of oxygen following cardiac arrest. These differences in experimental conditions may help explain why, in our patients, neural responses to complex auditory stimuli of environmental sounds were preserved. Testing auditory responses during anesthesia has certainly the advantage of evaluating preserved auditory functions in healthy controls under controlled levels of sedation. Future studies in anesthetized subjects could reveal complementary evidence to what we show in comatose patients in the present study.

Our results are more directly comparable with studies in animal models, where the neural correlates of auditory categorical discrimination have been revealed both in awake (Poremba et al., 2004; Recanzone, 2008; Perrodin et al., 2011) and anesthetized preparations (Rauschecker et al., 1995; Petkov et al., 2006, 2008). In particular, Petkov et al. (2008) showed an enhanced activation in response to conspecific vocalizations in comparison to other natural sounds in a number of regions of the monkey brain located mostly in the right primary and posterior parabelt fields as well as in bilateral anterior fields. The same experiment in anesthetized monkeys revealed the existence of a region in the anterior temporal lobe preferentially responding to conspecific vocalizations.

Our results affect our current understanding of auditory processing of environmental sounds by showing that auditory responses to environmental sounds can be classified even when subjects are unconscious. This complements recent findings with regard to the existence of a possibly specific activation to voices in different experimental contexts as a function of task demand (Capilla et al., 2013). Our study likewise extends these findings by showing that this invariance with respect to task instruction could instead be the result of an automatic mechanism that subtends the electrophysiological response to voices even when no perceptual process could take place. In addition, we showed that results were significant at the group level only for the comparison between vocalizations, suggesting that this automatic response might survive without consciousness only for specific types of auditory categories which are likely to be more relevant for the patients. Indeed, the voice category carries important emotional information, supports communication, and is crucial for establishing a speaker's identity.

Our study relates to previous literature focused on semantic processing in comatose patients. Most previous studies included patients in later stages of coma (>4 days after coma onset in Daltrozzo et al., 2009) and used linguistic material (Fischer et al., 2008; Qin et al., 2008; Rama et al., 2010). Moreover, no study had been performed on patients treated with TH and while patients were under sedation; conditions which allow us to conclude that all these patients were, at least in the first recording, totally unconscious. Indeed, our patients were acutely treated with sedative drugs (benzodiazepines together with opioid derivates) during the first recording, following a severe encephalopathy because of cardiac arrest, which occurred very shortly before the study (hours, to a few days). For clinical purposes, all these patients were diagnosed by neurologists and neuro-intensivists experienced in evaluating coma. The Glasgow Coma Scale and the FOUR score were assessed in all our patients and demonstrated severe consciousness impairment. Importantly, the FOUR coma scale has proven sensitive to detecting a minimal level of consciousness even in intubated patients (Bruno et al., 2011). In general, we cannot formally rule out that some of these patients might have been in a minimally conscious state (MCS), but this seems very unlikely, given the kinetics of assessment in relationship to the initial brain insult.

In previous research involving comatose patients, no studies have systematically checked for low-level acoustic differences between sound categories. Specifically, differences in the time-frequency representations of sounds can in and of themselves suffice to elicit differential responses at the level of primary auditory cortices. Despite these differences, the percentage of patients exhibiting accurate discrimination in our study (58 and 53%, respectively, for the comparison of vocalizations and that of sounds from living vs. man-made sources) is similar to what has been reported in previous studies based on linguistic material (Fischer et al., 2008; Daltrozzo et al., 2009) where less than 50% of the patients showed evidence of auditory discrimination. In the present study, we controlled groups of sound categories in terms of acoustic properties exemplified by the HNR and in their spectro-temporal representation. At no latency did we find any evident acoustic difference in the contrast between animal vs. human vocalizations or between living and man-made sounds (Figure 1). Based on these results, we can conclude that the categorical discrimination as measured by EEG could not be explained by an obvious difference at the level of the acoustic features of the sounds. However, the analyses we have carried out up to now do not exclude that combinations of specific acoustic attributes of each category could partially explain the significant decoding results. This aspect remains at the center of an open and active debate (Leaver and Rauschecker, 2010; Giordano et al., 2013). In some previous studies, the impact of spectro-temporal features on auditory processing of environmental sounds was controlled by investigating the neural correlates of the “scrambled” version of the sounds (Belin et al., 2000; Fecteau et al., 2004; Kriegstein and Giraud, 2004; Plourde et al., 2006). The use of scrambled sounds allows one to investigate the neural correlates of sounds with spectro-temporal characteristics very similar to the original ones while losing their semantic content. In this study we did not consider these types of auditory stimuli, and cannot fully exclude the impact of some combinations of the acoustic features on the significant decoding.

### AEPs in control subjects and comparison to patients

The decoding method was also applied to age-matched healthy controls, and revealed that four out of eight subjects exhibited significant categorization either between vocalizations and/or between sounds from living and man-made objects. Significant differential responses to animal/human vocalizations and living/man-made sounds were also evident when decoding response type across the 10 subjects. However, the results obtained in the healthy controls are not directly comparable to previous EEG studies based on the same type of analysis of responses to environmental sounds (De Lucia et al., 2012). First, our controls were age-matched to the comatose patients and therefore older (mean age = 57.6 ± 1.2) than those from previous literature (Murray et al., 2006; Simanova et al., 2010; De Lucia et al., 2012). Moreover, in contrast to previous studies (Levy et al., 2003; Murray et al., 2006; Simanova et al., 2010; De Lucia et al., 2012), the subjects were not performing an active task, but were listening passively to the sounds. Furthermore, since we applied the same clinical protocol as for the comatose patients, our EEG involved only 19 electrodes, whereas previous literature acquired EEG data with at least 64 electrodes (Simanova et al., 2010; De Lucia et al., 2012). Despite these differences, we could obtain significant results in a subset of the controls and accurate decoding at the group level with similar AUC values similar to a previous study based on a shorter version of the stimuli used in the present study (De Lucia et al., 2012). The periods of differential activity for both comparisons were nevertheless observed later (~200 ms post-stimulus onset) than what reported before (112 ms post-stimulus onset in (De Lucia et al., 2012); a difference which is possibly explained by the relatively older population of the present study. The periods of differential activity observed in patients exhibit a low degree of consistency within each group recorded during TH and NT and in comparison to controls (Figures 3A,B). Even though this heterogeneity prevents us to derive general conclusions about the possible mechanisms underlying the categorical discrimination in patients, we would notice that in all the comparisons and irrespective of being recorded during TH or NT, the earliest of the discriminative time periods appear before 300 ms, latencies which are compatible with an automatic process (Dehaene and Changeux, 2011).

### Comparison with previous clinical studies

In our study, successful auditory categorization during early stages of post-anoxic coma does not appear to be directly linked to a patient's likelihood of survival (Tables 2, 3), challenging the notion that the integrity of auditory processing is in itself predictive of outcome, at least when based on simpler auditory discrimination such as the kind observed during MMN paradigms (Kane et al., 1993; Guerit et al., 1999; Fischer et al., 2004; Naccache et al., 2005; Wijnen et al., 2007). These findings provide new insight about the existence of specific responses to auditory stimuli allowing at least a gross discrimination between auditory categories.

It is also worth noting that the vast majority of the patients showed an evoked response to sounds as revealed by the TCT, irrespective of whether they provided evidence of auditory discrimination. However, this significant evoked response did not always occur at the typical latency of the N100 component of a classical auditory evoked response nor with typical voltage topographies at these latencies (see Figure 2 for an exemplar patient). This observation underscores the advantage of applying a pattern recognition analysis that can reveal stimulus-related information without *a priori* criteria of inclusion as it has often been the case in previous literature on auditory processing in comatose patients (Fischer et al., 1999a, 2004). Previous studies relying on the existence of a classic N100 response as a pre-inclusion criterion produced a systematic rejection of about 30% of the patients (Fischer et al., 1999b, 2004); a bias which could influence the results and their interpretation. In addition, the single-trial decoding we applied here does not require the selection of specific time windows or latencies at which the effect of interest is pre-supposed to occur. Rather, it can discover any kind of spatio-temporal pattern localized in time or unfolding along several time periods of differential activity (Figures 2, 3). This aspect makes it quite different from other decoding methods commonly used in neuroimaging and cognitive studies, which usually explore the decoding performance over predefined time windows or over the whole trial (Philiastides and Sajda, 2006; Simanova et al., 2010; De Vos et al., 2012; Hausfeld et al., 2012). Integrating stimulus-related information that unfolds over multiple time periods of differential activity allows distributed patterns of activations in the discrimination between auditory categories to be taken into account, and leads to a more flexible strategy for optimal decoding performance.

## Conclusions and future directions

We have provided evidence that robust discrimination of environmental sounds can be achieved without awareness, and even in challenging clinical conditions such as those experienced by comatose patients during the acute phase.

However, it is worth noting that this study focuses on one type of coma, in very specific experimental contexts (half of the recordings were conducted under hypothermia and sedation). Because of the specific focus of the study, at present we cannot derive general conclusions about the extent of intact auditory processing and discrimination during acute coma and irrespective of the influence of sedation and hypothermia. Indeed, previous evidence based on classical MMN paradigms (Tzovara et al., 2013) has already suggested that hypothermia can aid the detection of auditory evoked responses, as shown by the relatively higher auditory discrimination exhibited by comatose patients under TH with respect to normal temperature (though this was not as clearly evident in the present study). Possible influences of TH on auditory responses include the reduction of physiological background noise, thereby allowing more reliable measurement of the evoked response to incoming stimuli (Madhok et al., 2012).

Future research will be necessary to further explore the neural correlates of auditory discrimination, with higher density electrode montages that in turn allow for more extensive investigation of the underlying neural sources in patients treated and not treated with TH. Little is known about the degree and nature of intact neural functions at this low temperature and under sedation, despite the clinically-demonstrated beneficial role of TH for improving recovery and neurological outcome (Bernard et al., 2002; Oddo et al., 2006; Rossetti et al., 2010). Additional research on the implicit categorization of environmental sounds could also benefit from focusing on healthy subjects under varying levels of sedation, allowing for a controlled experimental setup of the degree of consciousness. Indeed, hypoxia can affect the brain structures to different degrees of severity (Miyamoto and Auer, 2000), which we did not take into account in the present study. Other possible directions of research will focus on the discrimination of other auditory categories in the absence of awareness, including musical instruments and other tools, in an attempt to understand the minimum requirement for processing sound-related concepts in clinical (Trumpp et al., 2013b) and healthy populations (De Lucia et al., 2009; Hoenig et al., 2011).

## Author contributions

Athina Tzovara, Andrea O. Rossetti, Marzia De Lucia designed research; Natacha Cossy, Alexandre Simonin performed research; Natacha Cossy, Athina Tzovara, Marzia De Lucia analyzed data; Natacha Cossy, Athina Tzovara, Andrea O. Rossetti, Marzia De Lucia wrote the paper; and Andrea O. Rossetti and Marzia De Lucia provided funding.

## Conflict of interest statement

The authors declare that the research was conducted in the absence of any commercial or financial relationships that could be construed as a potential conflict of interest.
