# Supplementary material for: Robust discrimination between EEG responses to categories of environmental sounds in early coma
Source: Front Psychol. 2014 Feb 25;5:155. doi: 10.3389/fpsyg.2014.00155 (PMC3933775; doi:10.3389/fpsyg.2014.00155)
Supplement: Supplementary file 1 [file Presentation1.PDF]

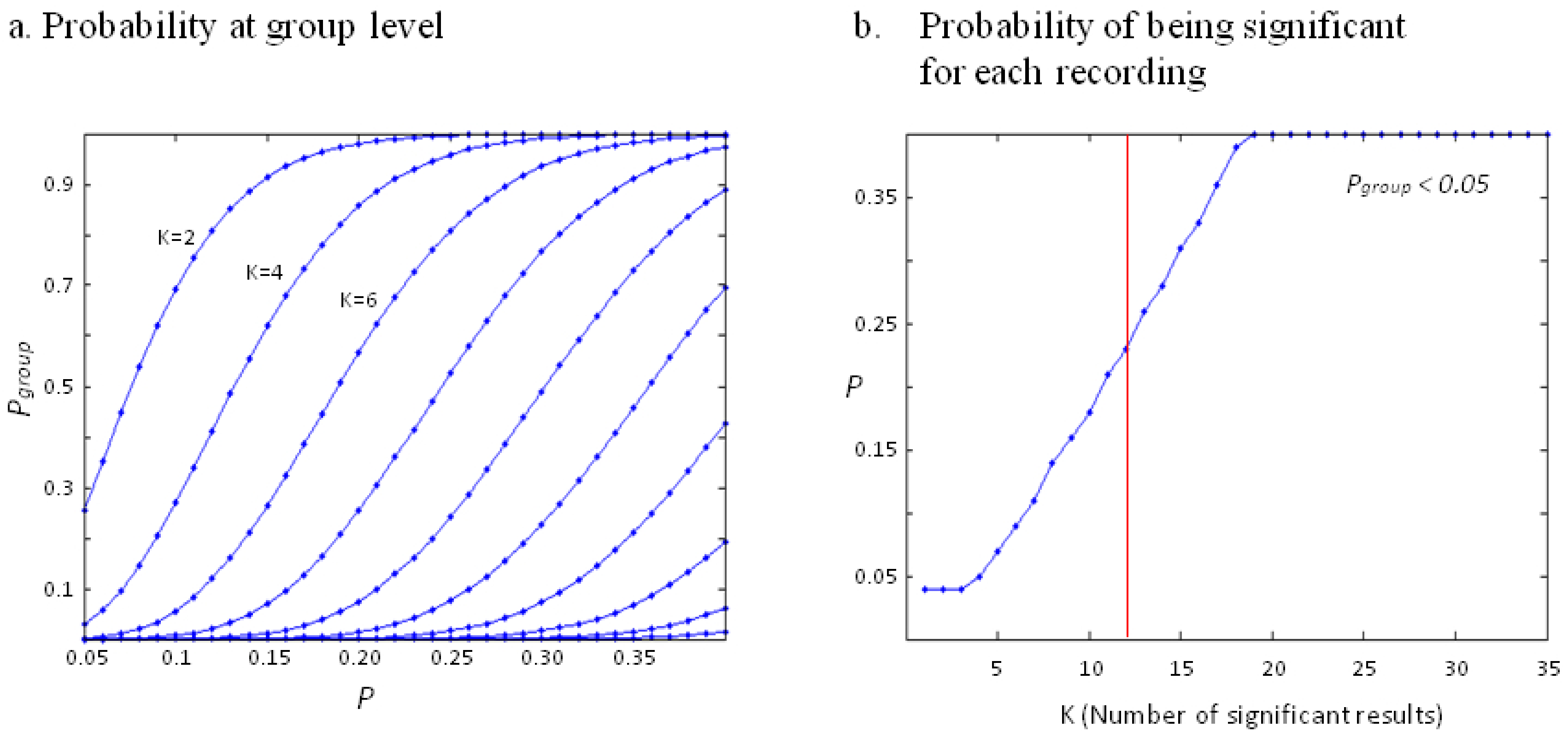

Supplemental Figure. a. Probability of significant results at group level, including a total of 35 recordings as a function of the significance, *P*, for each recording. Each curve corresponds to a value of significant results among the 35. By increasing the value of *p*, the total number of significant results, k, has to increase in order to keep the P_{group} below a certain threshold (i.e. 0.05). **b.** Value of significance for each recording at the threshold of Pgroup < 0.05 and as a function of the total number of significant recordings. In our case, when decoding EEG single-trial in response to vocalization types, we obtained that the validation results were better than chance with a probability of p<0.28 in 12 recordings (red line). Our results were therefore significant at group level.
